# Impact of noise on development, physiological stress and behavioural patterns in larval zebrafish

**DOI:** 10.1038/s41598-021-85296-1

**Published:** 2021-03-23

**Authors:** Rafael A. Lara, Raquel O. Vasconcelos

**Affiliations:** 1 Institute of Science and Environment, University of Saint Joseph, Macao S.A.R., China; 2 Departamento de Biología, Universidad de Sevilla, Seville, Spain

**Keywords:** Embryology, Environmental impact, Freshwater ecology

## Abstract

Noise pollution is increasingly present in aquatic ecosystems, causing detrimental effects on growth, physiology and behaviour of organisms. However, limited information exists on how this stressor affects animals in early ontogeny, a critical period for development and establishment of phenotypic traits. We tested the effects of chronic noise exposure to increasing levels (130 and 150 dB re 1 μPa, continuous white noise) and different temporal regimes on larval zebrafish (*Danio rerio*), an important vertebrate model in ecotoxicology. The acoustic treatments did not affect general development or hatching but higher noise levels led to increased mortality. The cardiac rate, yolk sac consumption and cortisol levels increased significantly with increasing noise level at both 3 and 5 dpf (days post fertilization). Variation in noise temporal patterns (different random noise periods to simulate shipping activity) suggested that the time regime is more important than the total duration of noise exposure to down-regulate physiological stress. Moreover, 5 dpf larvae exposed to 150 dB continuous noise displayed increased dark avoidance in anxiety-related dark/light preference test and impaired spontaneous alternation behaviour. We provide first evidence of noise-induced physiological stress and behavioural disturbance in larval zebrafish, showing that both noise amplitude and timing negatively impact key developmental endpoints in early ontogeny.

## Introduction

Anthropogenic noise has increased unprecedentedly in the last century both on land and underwater, being considered a global environmental pollutant by international legislation^1–3^. Noise pollution derives mostly from traffic, industry, resource extraction, construction and recreational activities, and it is expanding in time and space with subsequent negative impacts on human health and wildlife^4–6^. Besides a significant increase in overall sound levels, anthropogenic noise sources add new sounds into the environment that differ greatly in spectral composition and duty cycle from the natural soundscapes^7,8^. For instance, shipping and recreational boats add a broadband noise component into the overall aquatic acoustic scene that lasts much longer compared to pile driving and seismic air guns that generate impulsive short-lasting low frequency sounds^9,10^.

Repeated or chronic exposure to increased noise levels can affect how animals respond to this stressor due to mechanisms of habituation and sensitization that rely on either augmented or decreased tolerance, respectively^11–15^. Shifts in tolerance to noise exposure, or to any other stressor, typically depend on duration, intensity and time regime of exposure^11,16^, hence identifying patterns with less impact is paramount for defining sustainable noise management and mitigation strategies. Noise is known to cause a myriad of detrimental effects in various taxa including auditory impairment^17–20^, impaired development^21,22^, heightened physiological stress^8,19,23,24^ and behavioural disturbance^7,19,20,23,25^, thus posing unprecedented risks on species survival, biodiversity and ultimately on ecosystems health.

Considering how fast aquatic soundscapes are changing^26^, it is fundamental to develop research on how different noise regimes affect development, physiology and behaviour of fish, as they are key components of most aquatic ecosystems^27^. An increasing amount of studies are showing the negative effects of anthropogenic noise on fish species, including lowered survivability^28^, impaired growth^13,29^, increased physiological stress^30,31^, compromised hearing^32,33^, and behavioural disturbance^34,35^. The majority of these studies, however, focused on the effects of short-term exposure to intense noise amplitudes, and most of the impact is likely to derive from less noticeable mild noise levels and repeated/chronic exposure^36^. This type of exposure can introduce modifications on how species respond to the stressor due to changes across time and cumulative effects, as well as impose driving evolutionary pressures^27,37,38^.

Only few studies have evaluated long-term noise effects on animals^19,20,39,40^ and particularly in early ontogeny^41,42^, a critical period for development and establishment of phenotypic traits^43^. In fish, very scarce information exists on how chronic exposure to this environmental stressor impacts species in the adult stage^31,44,45^ and the effects on larval survivability^46^, development and behaviour^13,47^, and physiological stress^48^. So far, only one study evaluated how prolonged noise exposure varying in regularity can impact fish in early development^13^. This study demonstrated that two days of either regular or random noise may affect growth, while regular regime also led to faster yolk sac consumption and altered body condition in larval Atlantic cod *Gadus morhua*. Further research is needed regarding the impact of chronic noise exposure on early development using model organisms that allow for integrative studies combining morphological, physiological, behavioural and genetic approaches, in order to unravel coping mechanisms of acoustic stress.

The zebrafish *Danio rerio* has become a reference vertebrate model in the fields of developmental biology^49^, ecotoxicology^50,51^ and hearing research^52^. This species has been further used to test the impact of noise exposure on lateral line hair cells^53^ and auditory-evoked escape responses^54^, but the effects of acoustic stress on development, physiology and anxiety-related behaviors remain unknown.

In the present study we performed a split-brood experiment to test the effects of chronic exposure to noise of different amplitude levels and temporal patterns (random intermittent regimes simulating shipping activity) on development, physiological stress and behavioural traits in larval zebrafish. We used ecologically-relevant mild noise levels, such as 130 and 150 dB re 1 μPa, which are representative of boat noise and amplitudes found in zebrafish housing systems^55^.

## Results

### Hatching, growth and mortality

The noise treatments used in this study did not significantly affect the hatching rate of zebrafish embryos (comparison between noise levels—control, 130 dB (CN_130_) and 150 dB (CN_150_): F_(2, 38)_ = 2.80, *p* = 0.074; and temporal variations—control, CN, IN1, IN2, IN3: F_(4, 47)_ = 0.76, *p* = 0.25), nor cause any obvious developmental abnormalities. Embryos started hatching at 2 dpf (i.e. control: 10–11%; CN_130_: 9–10%; CN_150_: 7–8%, without differences between treatments and by 3 dpf all viable specimens hatched. Furthermore, changes in both noise level and temporal patterns did not affect the total length of the larval zebrafish measured at 3 dpf (noise levels: F_(2, 131)_ = 0.32, *p* = 0.655; temporal variation: F_(4, 147)_ = 5.19, *p* = 0.66) nor at 5 dpf (noise levels: F_(2, 131)_ = 0.17, *p* = 0.735; temporal variation: F_(4, 143)_ = 0.64, *p* = 0.83).

**Figure 1:**
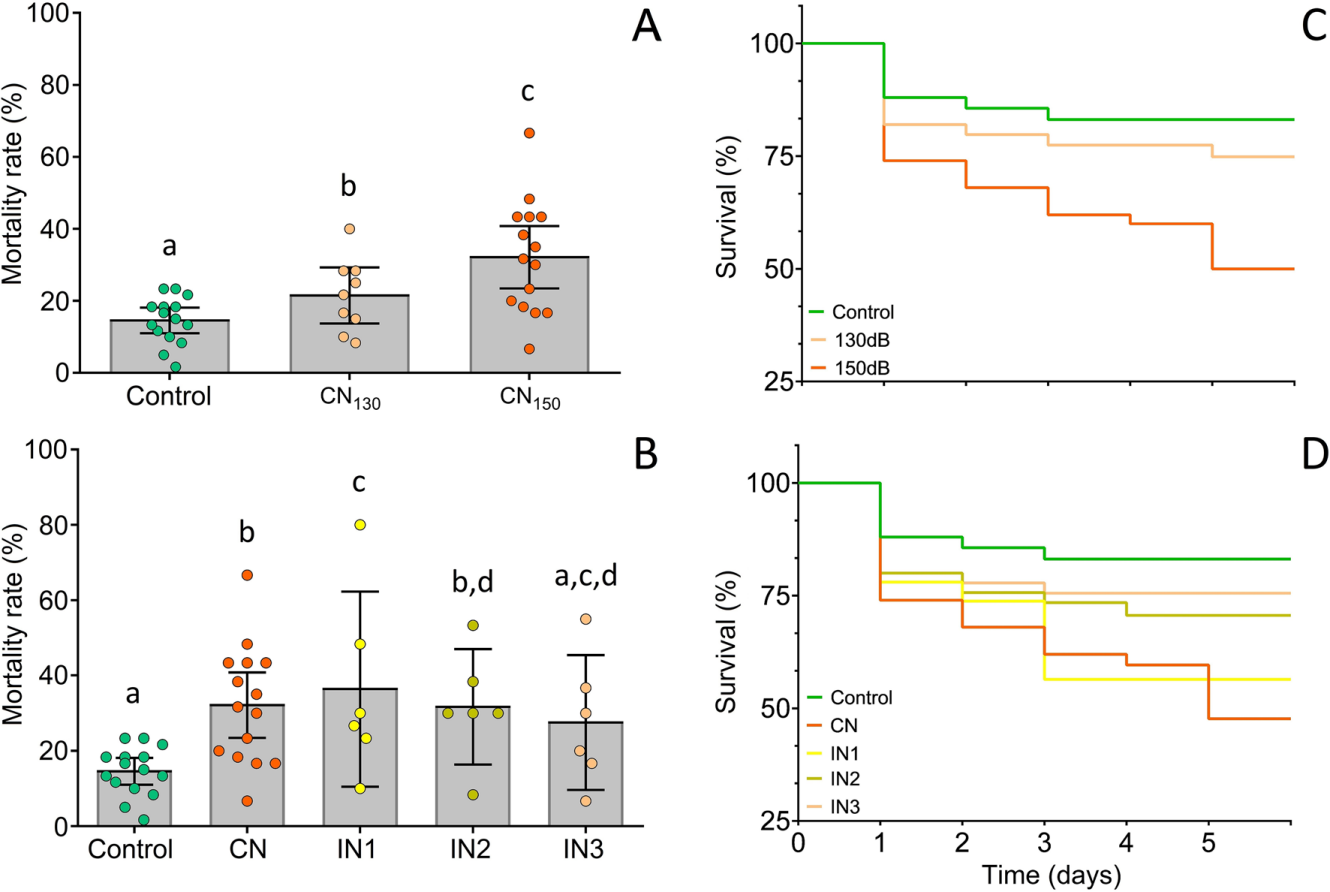
Comparison of mean mortality rate between treatment groups (larval zebrafish up to 5 days post fertilization) exposed to (**A**) continuous noise at different amplitudes (F_(2, 38)_ = 8.71, *p* < 0.001) and (**B**) varying noise temporal patterns (F_(4, 47)_ = 3.78, *p* = 0.005). Kaplan–Meier percentual survival plot of zebrafish larvae throughout the 5-days of chronic acoustic treatments of (**C**) increasing noise amplitudes and (**D**) varying temporal patterns. Control- silent conditions, CN- continous noise at either 130 (CN_130_) or 150 dB re 1 μPa (CN_150_), IN- intermittent regime with short (IN1), medium (IN2) and long noise segments (IN3). Error bars represent 95% confidence intervals. Different letters indicate statistically significant differences between specific groups based on post hoc tests.

However, an effect of noise exposure on mortality was observed throughout the acoustic treatments for specimens under continuous noise (CN) (F_(2, 38)_ = 8.71, *p* < 0.001), with CN_150_ causing a significant increase compared to CN_130_ (*p* = 0.036) and control (*p* < 0.001) (Fig. 1A,C). Variation in the timing of acoustic disturbances also induced an increase in mortality in all the noise exposed groups compared to control (F_(4, 47)_ = 3.78, *p* = 0.005) (Fig. 1B,D). Short (IN1) and medium (IN2) noise periods of intermittent treatments induced higher mortality rates similar to CN (IN1 vs. control: *p* = 0.008; IN2 vs. control: *p* = 0.015), while the treatment presenting long noise segments (IN3) did not induce significant mortality compared to control (*p* = 0.066) (Fig. 1B,D).

### Physiological stress indicators

**Figure 2:**
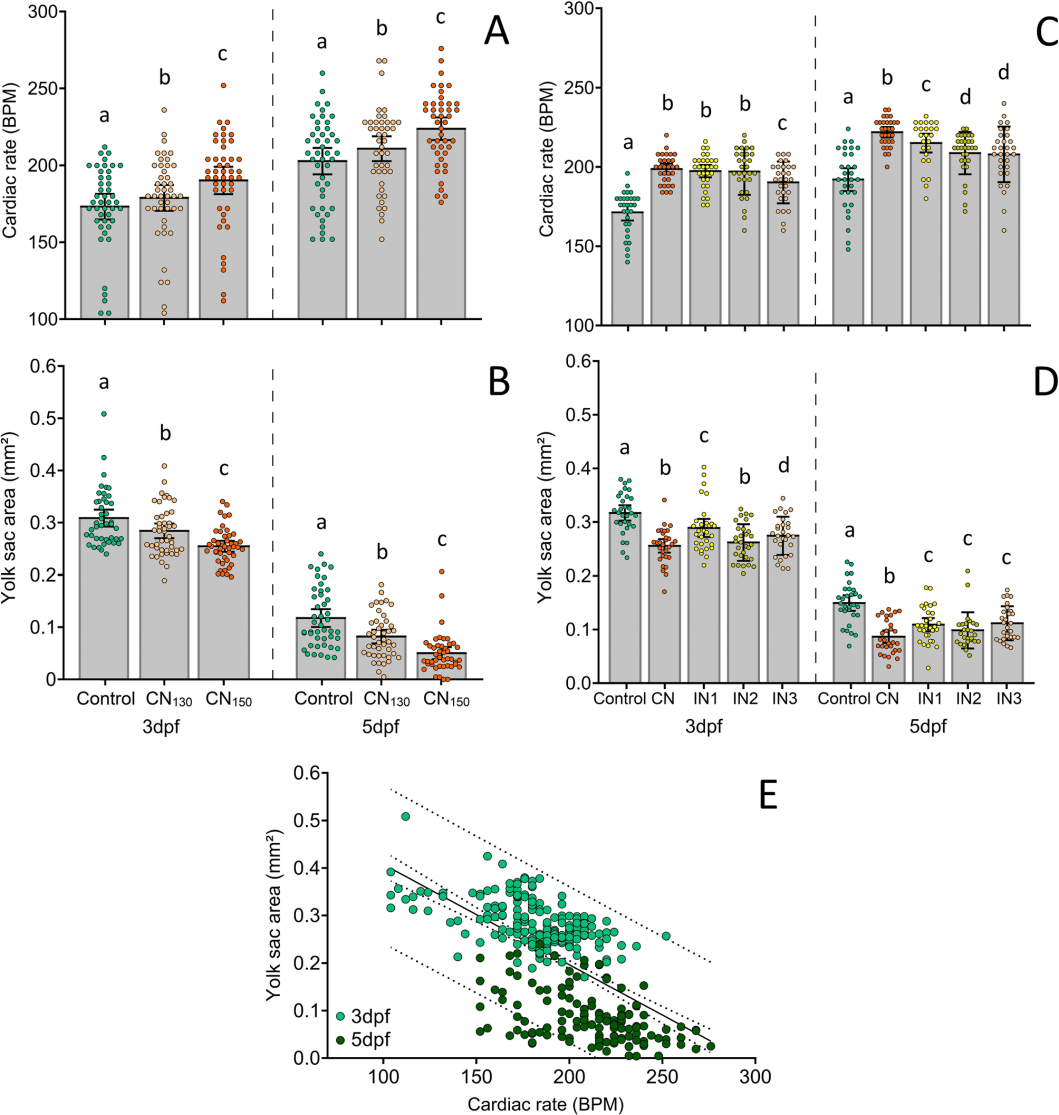
Comparison of mean cardiac rate and yolk sac area of larval zebrafish with 3 and 5 dpf (days post fertilization) exposed to different noise amplitudes (**A** and **B**) and temporal patterns at 150 dB re 1 μPa (**C** and **D**). Increasing noise amplitudes induced heightened cardiac rate at 3 dpf (F_(2, 134)_ = 4.20, *p* = 0.004) and 5 dpf (F_(2, 133)_ = 7.17, *p* = 0.002), as well as a decrease in yolk sac (3 dpf—F_(2, 136)_ = 11.96, *p* < 0.001; 5 dpf—F_(2, 134)_ = 16.59, *p* < 0.001). Cardiac rate was further affected by noise temporal variation at 3 dpf (F_(4, 146)_ = 25.36, *p* < 0.001) and 5 dpf (F_(4, 143)_ = 15.50, *p* < 0.001), as well as the yolk sac 3 dpf (F_(4, 145)_ = 13.79, *p* < 0.001) and 5 dpf (F_(4, 143)_ = 12.19, *p* < 0.001). Error bars represent 95% confidence intervals. Different letters indicate statistically significant differences between specific groups based on post hoc tests. (**E**) Correlation between cardiac rate and yolk sac size (R =  − 0.61, N = 370, *p* < 0.001) from both 3 and 5 dpf. Solid line—best-fitted line; inner dashed lines—standard error of the mean; outer dashed lines—95% confidence interval.

Cardiac rate, yolk sac consumption and cortisol levels increased significantly with increasing noise level, which clearly indicated higher physiological stress. The cardiac rate of larval zebrafish at 3 dpf was about 173 ± 30 bpm (mean ± SEM, standard error of the mean; bpm, beats per minute) for control and increased to 191 ± 60 bpm after playback of CN_150_, whilst at 5 dpf it was around 203 ± 40 (control) and increased to 224 ± 50 bpm after CN_150_ treatment. A significant increase was verified with increasing noise level in both 3 dpf (F_(2, 134)_ = 4.20, *p* = 0.004) and 5 dpf larvae (F_(2, 133)_ = 7.17, *p* = 0.002), with CN_150_ causing the highest differences compared to control (*p* < 0.001) (Fig. 2A).

Similarly, the yolk sac consumption increased significantly with increasing noise level at both 3 dpf (F_(2, 136)_ = 11.96, *p* < 0.001) and 5 dpf larvae (F_(2, 134)_ = 16.59, *p* < 0.001), with CN_150_ also inducing the highest differences compared to control (*p* < 0.001) (Fig. 2B).

Variation in the timing of the acoustic disturbances caused different effects on both cardiac rate and yolk sac consumption, suggesting that noise temporal regime is important to regulate physiological stress and depletion of embryonic endogenous energy reserves. The cardiac rate was significantly affected by noise time variations at both 3 dpf (F_(4, 146)_ = 25.36, *p* < 0.001) and 5 dpf (F_(4, 143)_ = 15.50, *p* < 0.001), with continuous noise causing the highest impact compared to intermittent treatments at 5 dpf (*p* < 0.001) (Fig. 2C). IN3 consistently induced the lowest impact among intermittent regimes at both 3 and 5 dpf compared to control (*p* = 0.009 and *p* = 0.046, respectively). Additionally, significant differences in the yolk sac consumption were found between these noise treatments at 3 dpf (F_(4, 145)_ = 13.79, *p* < 0.001) and 5 dpf (F_(4, 143)_ = 12.19, *p* < 0.001) (Fig. 2D). CN induced the highest yolk sac consumption compared to intermittent noise groups at 5 dpf (*p *< 0.001).

Overall, the cardiac rate was negatively correlated with yolk sac size (R =  − 0.61, N = 370, *p* < 0.001), meaning that individuals with higher cardiac rate consumed their energy reserves faster (Fig. 2E).

**Figure 3:**
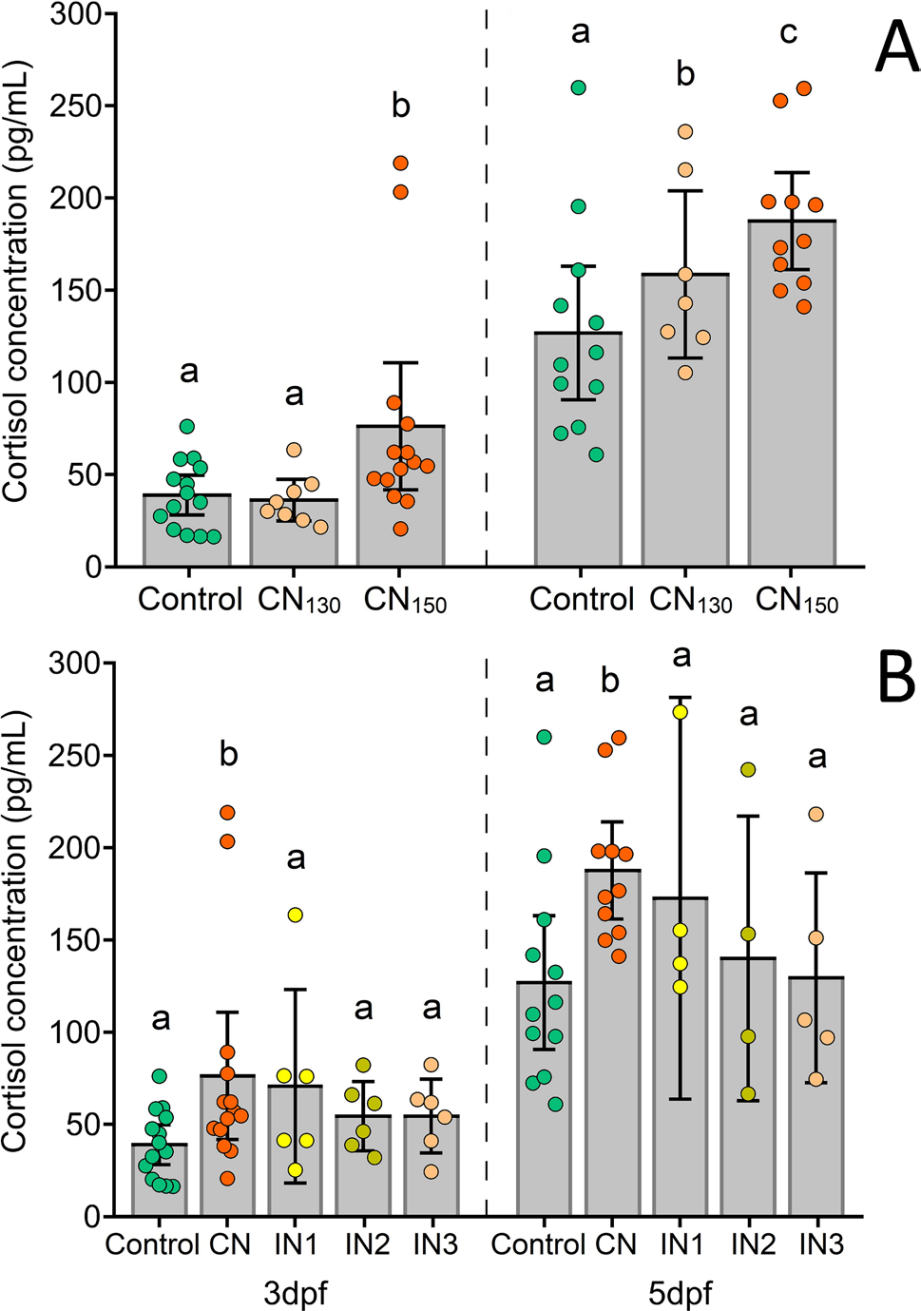
Whole-body cortisol levels from larval zebrafish exposed to (**A**) continuous noise at different amplitudes (3 dpf: F_(2, 35)_ = 4.84, *p* = 0.014; 5 dpf: F_(2, 29)_ = 4.37, *p* = 0.023) and (**B**) varying noise temporal patterns at 150 dB re 1 μPa (3 dpf: F_(4, 45)_ = 2.23, *p* = 0.082; 5 dpf: F_(4, 35)_ = 2.65, *p* = 0.052). Control- silent conditions, CN- continous noise at either 130 (CN_130_) or 150 dBre 1 μPa (CN_150_), IN- intermittent regime with short (IN1), medium (IN2) and long noise segments (IN3). Error bars represent 95% confidence intervals. Different letters indicate statistically significant differences between specific groups based on post hoc tests.

Noise-induced physiological stress was confirmed through whole-body cortisol quantification. Cortisol levels increased significantly with noise amplitude at both 3 dpf (F_(2, 35)_ = 4.84, *p* = 0.014; CN_150_ higher than control *p* = 0.010) and 5 dpf (F_(2, 29)_ = 4.37; *p* = 0.023; both CN_130_ and CN_150_ higher than control *p* = 0.016 and *p* = 0.004 respectively) (Fig. 3A). Variation in the noise time regime caused changes in cortisol, however, they were not statistically significant—3dpf (F_(4, 45)_ = 2.23, *p* = 0.082); 5 dpf (F_(4, 35)_ = 2.65, *p* = 0.052) (Fig. 3B).

### Behavioural patterns

**Figure 4:**
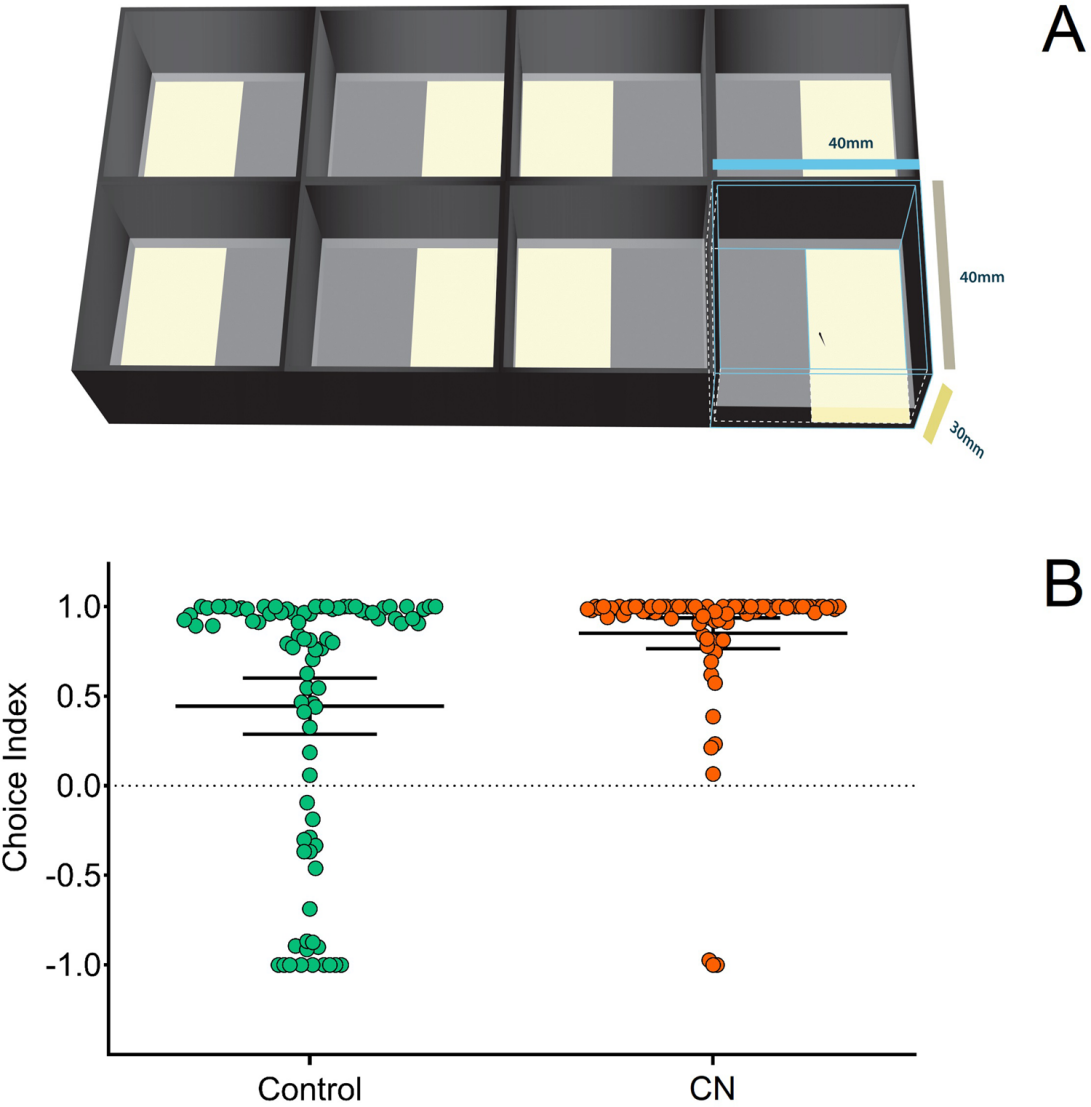
(**A**) Light/dark preference assay consisting of squared plastic compartments (each 40 mm width × 40 mm length × 30 mm height) divided into two equal sized areas with distinct bottom illumination (transparent/bright versus opaque/dark). The apparatus was placed on top of a LED panel (~ 7000 lx). Each compartment was filled with 10 ml water and a single larval zebrafish (5 dpf) was placed in the middle of the arena and recorded for 5 min. (**B**) Choice index for larva exposed to continuous noise (150 dB re 1 μPa) versus control conditions (U_(2, 173)_ = 5341.50, *p* < 0.001). Choice index was calculated as: (Time in dark–Time in light)/(Time in dark + Time in light). Individual data are presented as scatter plots and bars depict mean ± 95% confidence intervals. Illustration was created using Adobe Illustrator CC 2020 version 24.0.2 for Windows, Adobe Inc., CA, USA and Blender version 2.80 for Windows, Blender Foundation, AMS, EU.

In order to assess potential changes at the behavioural level, 5 dpf larvae exposed to 150 dB continuous noise (CN_150_) were further tested using an anxiety-related light/dark preference test (Fig. 4A). Specimens exposed to the acoustic stressor exhibited stronger dark avoidance or scotophobia, as measured based on a choice index (U_(2, 173)_ = 5341.50; *p* < 0.001) (Fig. 4B).

**Figure 5:**
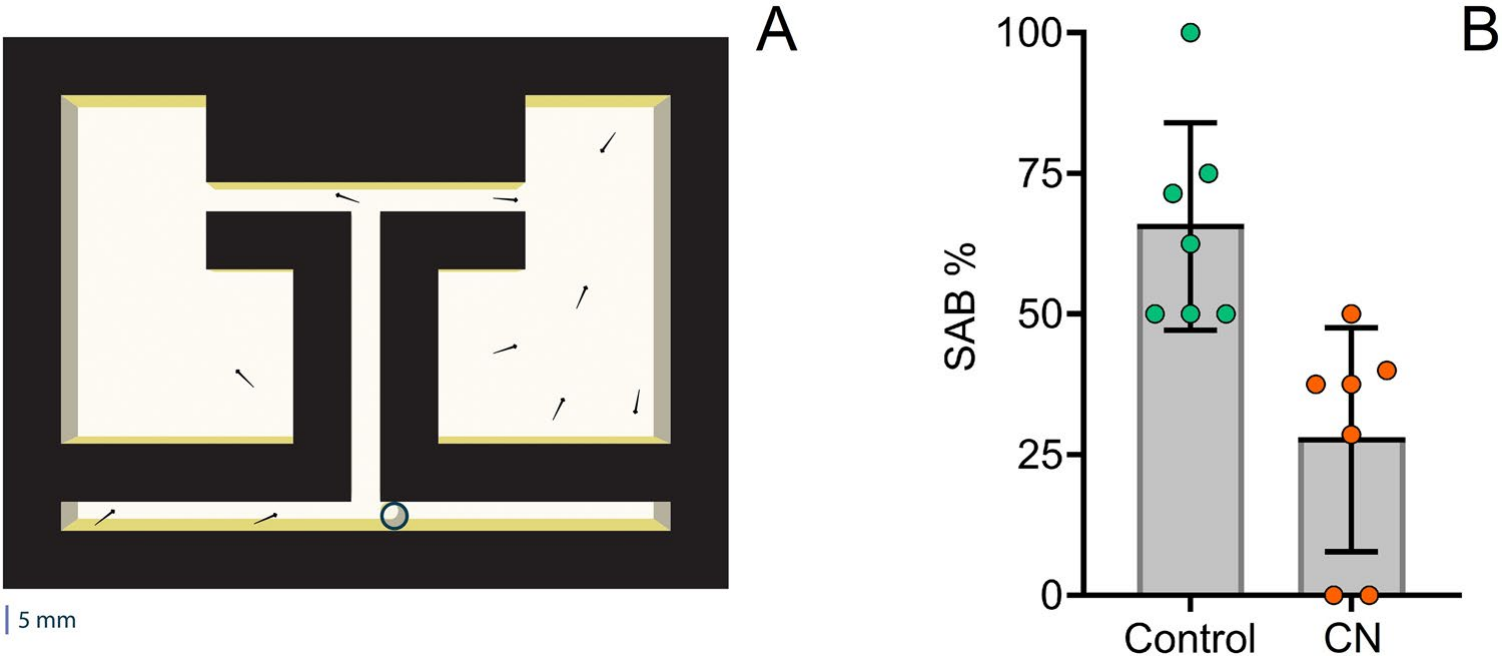
(**A**) Spontaneous Alternation Behaviour (SAB) assay with bottom illumination to test exploratory swimming and spatial memory. The starting arms can be used alternatively (a plastic tube blocks the entrance to the opposite arm) and converge into a perpendicular main arm that leads to a choice of alternation or same side arm. These arms lead to distinct pools of 19.50 mm^2^. (**B**) Comparison of SAB in 5 dpf under continuous noise at 150 dBre 1 μPa (CN) and control conditions (t_(113)_ =  − 4.08, *p* < 0.001). From a total of 180 tested larvae, 115 successfully showed alternation behavior (entered the opposite side pool) within the 10-min recording. Individual data are presented as scatter plots and bars depict mean ± 95% confidence intervals. Illustration was created using Adobe Illustrator CC 2020 version 24.0.2 for Windows, Adobe Inc., CA, USA and Blender version 2.80 for Windows, Blender Foundation, AMS, EU.

Larval zebrafish (5 dpf) were also tested regarding exploratory behaviour using the Spontaneous Alternation Behaviour (SAB) assay (Fig. 5A), which describes the tendency of animals to alternate their turn direction in consecutive turns^56,57^. The SAB assay revealed that 70% of larvae reared under silent control conditions exhibited normal swimming alternation, as opposed to only 34% from the noise treatment group (CN_150_) (t_(113)_ =  − 4.08, *p* < 0.001) (Fig. 5B). The swimming patterns of these specimens were further investigated in an open field arena, which revealed a reduction in covered area for the noise-treated larvae (t_(89)_ = 7.33, *p* < 0.001).

## Discussion

To our knowledge this is the first study assessing the impact of noise exposure on larval zebrafish (*Danio rerio*), a reference model organism in ecotoxicology. We provide evidence of noise-induced physiological and behavioural disturbance in larval zebrafish, and demonstrate that both amplitude and timing of the acoustic stressor may impact key health-related endpoints in early ontogeny.

The acoustic treatments considered in the present study did not affect hatching or general development but higher noise levels (130 and 150 dB re 1 μPa) led to increased mortality (from 15% in control up to 33% at the highest sound level). Very limited information exists on the impact of noise on fish hatching success and survival. Banner and Hyatt^46^ found significant lethal effects on fish embryos (*Cyprinodon variegatus*, Cyprinodontidae) exposed to higher noise conditions in a tank system with two distinct acoustic zones, but no effect was detected at the post hatching stage (> 24 hpf). Bruintjes and Radford^58^ evaluated the impact of small motorboat noise (127 dB re 1 μPa RMS) on a cichlid fish (*Neolamprologus pulcher*) and did not find an effect on hatching success or fry survival.

Previous studies described mixed results in regards to the impact of noise on fish growth. Banner and Hyatt^46^ reported noise-induced decrease in larval growth during the first 11–15 days post-hatch, whereas Bruintjes and Radford^58^ described an absence of effects on body length or weight after 4 weeks post-hatch. Both Davidson et al.^59^ and Nedelec et al.^13^ found that increased noise levels significantly reduced development in different fish species (rainbow trout *Oncorhynchus mykiss* and Atlantic cod *Gadus morhua*), however this was followed by a catch-up growth at later stages.

In the present study, the intermittent treatment with many onsets of acoustic disturbance (IN1, characterized by 5–12 s noise and 1–120 s silence, about 15% total noise) caused higher mortality up to 33%, similar to continuous noise exposure and IN2 (with similar noise exposure to IN1 but extended silent periods of 1–10 min). This was significantly above baseline levels in control group (15%) and contrasted with the IN3 treatment with prolonged noise presentations (total 50% noise presence) but fewer onsets that caused 27% in mortality. The relevance of the timing of acoustic exposure has received limited attention but increasing evidence points towards a significant impact on development and physiological stress in different fish species^13,60^. Nedelec et al. (2015) reported no difference in body length of larval codfish exposed to either regular or random playback of ship noise (average 15 min noise playback/hour)^13^. The body width-length ratio, however, declined over the course of the study and the greatest decline was registered under regular noise treatment at 16 days post-hatch. These larvae were easier to catch in a predator-avoidance experiment, demonstrating that the timing of acoustic disturbance can impact survival-related measures during development. Additionally, Neo et al.^60^ assessed the impact of continuous versus intermittent regular noise (0.1 s noise plus 0.9 s silence) on seabass adults (*Dicentrarchus labrax, Moronidae*) and found that intermittent exposure resulted in slower normal behaviour recovery compared to continuous treatment.

The present study also found heightened cardiac rate, yolk sac consumption, and cortisol levels at both 3 and 5 dpf zebrafish treated with elevated noise levels, which is a clear indication of noise-induced physiological stress. The control cardiac rate values varied between 173 and 191 (3 and 5 dpf, respectively), which is comparable to prior studies on the same model organism^61–63^. We verified an increase of about 10% in both ontogenetic stages (3 dpf: from 173 to 191 bpm; 5 dpf: 203–224 bpm) under increased noise conditions (CN_150_). Simpson et al.^48^ and Jain-Schlaepfer et al.^64^ reported first evidences of higher cardiac activity due to anthropogenic noise in fish larvae. The authors found that the embryos (staghorn damselfish *Amblyglyphidodon curacao* and clownfishes *Amphiprion* spp.) increased their heart rate (up to 5%) in the presence of increased levels of shipping noise, and that the physiological impact depended on the engine type.

Increased cardiac activity represents an adrenergic stress response, which is typically responsible for activating metabolic pathways and mobilizing energy to cope with potential challenges^65–69^. In this case, the perceived challenge consisted of acoustic disturbance, which is not a life-threatening situation. Hence, the energy depletion due to acoustic stress might be detrimental to the embryos that could otherwise use it for survival–related developmental processes.

To our knowledge, the present study is the first to measure cardiac rate and related it with endogenous embryonic energy substrates (yolk sac size) in fish. We provide evidence that these variables are significantly correlated. In addition, we confirmed the heightened physiological stress in noise-exposed larvae by measuring their cortisol levels that were significantly above control groups. Chronic exposure to an environmental stressor may interfere with resource allocation from reserves maintenance to activation of the adrenal system, resulting in allostatic load^70^. Our study shows that larval zebrafish under noise exposure (CN_150_) consume their yolk sac 21% (at 3 dpf) and 58% (at 5 dpf) faster compared to baseline conditions. Considering that noise-exposed individuals showed similar development (body size) to control group, we predict that the increased yolk consumption is not being invested in a faster development but reflects additional survival costs to cope with acoustic stress. The increased mortality registered in the noise-treated groups further supports this hypothesis. Nedelec et al. (2015) also reported an effect of shipping noise on yolk sac consumption in larval codfish^13^. Larvae exposed to regular noise used their yolk sac 29% faster after 2 days of exposure and had a lower body width-length ratio after 16 days post-hatch compared to specimens raised in quieter ambient noise conditions. Other studies have used the yolk sac size of larval fish to assess the impact of environmental stress^71–74^, and likewise showed that variables like salinity, temperature, light conditions and even maternal stress, can lead to a significant impact on the yolk sac absorption rate and composition.

Our work provides first evidence of noise-induced increase in whole-body cortisol levels in a larval fish. Cortisol elevation due to environmental stress, such as salinity, temperature, light conditions, acidity, mechanical disturbance, has been previously observed in larval zebrafish. While measuring units vary between studies, cortisol levels are consistently within a range of 5–10 pg/larva for control and 200–250 pg/larva for stressed individuals (within 3-7 dpf)^75–79^. For instance, Bait et al.^78^ reported an elevation in cortisol/protein of about 65% under cold conditions, 40% under UV light and almost 100% under mechanical disturbance. Our study reported a similar increase in cortisol levels of up to 95% (3 dpf) and 47% (5 dpf) due to acoustic stress. 

Variation in the temporal patterns of intermittent treatments caused different effects on cardiac rate, yolk sac consumption, and nearly on cortisol levels (although not statistically significant), suggesting that time regime of the acoustic disturbance is important to down-regulate physiological stress. Overall, continuous noise exposure induced the highest cardiac rate and yolk consumption compared to intermittent treatments, whilst IN3 characterized by longer noise playback but also prolonged silent intervals caused less impact on these variables compared to control and other IN treatments. Moreover, cortisol levels were generally elevated in noise-exposed groups, and the highest level reached under continuous noise treatment. Although not significantly different, IN3 induced lower cortisol level compared to IN2. Similar to Nedelec^13^, we hypothesize that longer silent intervals during random disturbances allow for recovery, compensation and/or habituation in larval fish, and that the total duration of noise exposure is less crucial compared to the time regime adopted (number of onsets of acoustic disturbance and silent intervals).

Comparing the effects of noise between 3 and 5 dpf larvae, we can conclude that the impact was generally more pronounced at the later developmental stage. The 5 dpf larvae have remained chronically exposed to the acoustic treatments for a significantly longer period of time, which most likely resulted in higher cumulative effects. Although both stages are sensitive to sound, the inner ear saccule of 5 dpf is more sensitive to auditory stimuli^80^, and this may have contributed for higher acoustic stress. Future work should investigate the most sensitive window during embryonic development for acoustic stress, the consequences for the structure and function of auditory pathways, and associated metabolic costs.

The present study also investigated the impact of noise exposure (CN_150_) at the behavioural level in larval zebrafish. We focused on 5 dpf, a developmental stage when larvae acquire full motility, display active feeding and danger/predator avoidance, suggesting that simple neural circuitries for processing reward and aversion are already functional^81,82^. We tested the effect of CN_150_ treatment on 5 dpf larvae using the anxiety-related light/dark preference test, which has been widely used to test stress and anxiety in mammals^83^ and zebrafish^78,84–87^. Our results indicate increased darkness aversion (scotophobia) in noise-exposed larvae, suggesting that such environmental stressor elicits anxiety behaviour. Similarly, Bai et al.^78^ reported that heat, cold and UV treatment significantly enhanced darkness aversion in larval zebrafish. The authors also found that treatment with two anxiolytics with different pharmacokinetics (chlordiazepoxide, a GABAergic benzodiazepine and buspirone, a serotonin agonist) attenuated this behavior, which confirmed that such pattern was anxiety-driven. Future research should consider testing the effect of different anxiolytics on noise-treated zebrafish and evaluate light/dark preference at different development stages.

Finally, this study also showed that noise-exposed 5 dpf larvae (CN_150_) displayed impaired innate Spontaneous Alternation Behaviour compared to control individuals. Bögli et al.^88^ effectively established the presence of SAB in larval zebrafish (6 dpf) suggesting the presence of early mnestic capabilities. At this developmental stage (4–5 dpf), the hippocampal-like pallium develops^89^, and this brain structure is known to be related with navigation and spatiotemporal sensing in fishes^90,91^. Other studies have investigated the effect of acoustic experience in memory function. For instance, Cheng et al.^92^ reported impaired learning and memory capabilities in mice after exposure to white noise at 80 dB SPL for 2 h per day for a 6-week period, along with peroxidative damage and tau hyperphosphorylation in different brain structures including the hippocampus. Other authors have reported that environmental noise may induce stress-like effects in juvenile rats affecting spatial learning abilities, and that stress in developing rats can affect behavioral lateralization^93,94^.

Additionally, the overall locomotor activity of these specimens was further investigated and a significant reduction in covered area was observed in the noise-treated larvae. These results are in line with Bhandiwad et al.^54^, who investigated the effect of noise exposure (white noise at 20 dB re 1 ms^-2^) on larval zebrafish (5–7 dpf) and identified a significant decrease in total distance covered compared to control. Ongoing research aims to confirm whether impaired SAB resulted from spatial memory dysfunction and/or changes in the exploratory behaviour that are anxiety-driven or related to motor function. Additional work should evaluate the effect of different acoustic parameters (amplitude, temporal regime, spectral content) of noise exposure on behavioural patterns in larval zebrafish.

In summary, we provide first evidence of noise-induced physiological stress and behavioural disturbance in larval zebrafish, showing that increased noise amplitude and changes in the noise temporal regimes can induced higher cardiac rate and activate the adrenal system leading to increased cortisol levels and depletion of embryonic endogenous energy reserves. Intermittent sounds with short duration, such as those commonly found in aquatic systems with elevated traffic activity from small motor boats, speed boats and personal water crafts^95–99^, may have a stronger physiological and behavioural consequences on fish, including higher mortality, physiological stress and behavioural alterations, compared to regimes of similar or higher overall noise exposure but less number of noise onsets and longer silent periods.

Future studies are required to evaluate whether noise-induced energetic costs may result in carryover effects to subsequent life stages and fitness. We show that larval zebrafish can be established as a high-throughput platform for fast screening of the biological impact of acoustic disturbances at developmental, physiological and molecular levels. Furthermore, we highlight the importance of noise regularity and its consideration for noise management and mitigation strategies.

## Methods

### Fish husbandry and sampling

Zebrafish eggs were obtained from wild type adults (AB line) purchased from China Zebrafish Resource Center (CZRC, China) and reared at the research facilities of the University of Saint Joseph (Macao) following general guidelines for zebrafish housing and husbandry by Westerfield (2007)^100^. Stockfish were maintained in a standalone housing system (model AAB-074-AA-A, Yakos 65, Taiwan) with filtered and aerated water (pH balanced 7–8; 400–550 µS conductivity) at 28 ± 1 °C and under a 12:12 light/dark cycle.

For each experimental trial, eggs were collected within 2 hpf (hours post fertilization) from 2 to 6 breeding tanks (each tank containing about 10 females and 5 males). Collected eggs were mixed in a single Petri dish containing embryo medium and distributed into different groups of 50 specimens each. Depending on the experiment, either 3 or 5 groups were considered (see details below). This split-brood approach was adopted to minimize potential differences related to egg quality/viability. Each group was allocated to a different acoustic treatment tank that was randomly assigned for a specific treatment. The sample size (50 specimens per treatment group) was selected to ensure enough specimens for data collection in the multiple tests, and also based on previous observations of mortality rates.

Egg survivability was evaluated during the first 48 hpf after examination of the batches at a fixed time in the morning (between 10 and 11 am). Morphological and physiological data was consistently collected at the same time in the morning at two developmental stages, 3 and 5 dpf. These stages of development were selected since at 3 dpf embryos already have a functioning inner ear^101^, and at 5 dpf specimens already exhibit active feeding and sensorimotor behaviors, such as auditory-evoked escape responses, that are affected by noise exposure^54^.

All specimens that were used in experimental procedures were euthanized at the end in 300 mg/L of Tricaine Methanesulfonate (MS-222, Thermo Fisher Scientific INC, Massachussets, USA) based on the protocol by Strykowski and Schech (2015)^102^.

All experimental procedures complied with the ethical guidelines regarding animal research and welfare enforced at the Institute of Science and Environment, University of Saint Joseph, and approved by the Division of Animal Control and Inspection of the Civic and Municipal Affairs Bureau of Macao (IACM), license AL017/DICV/SIS/2016.

### Experimental design and acoustic treatments

**Figure 6:**
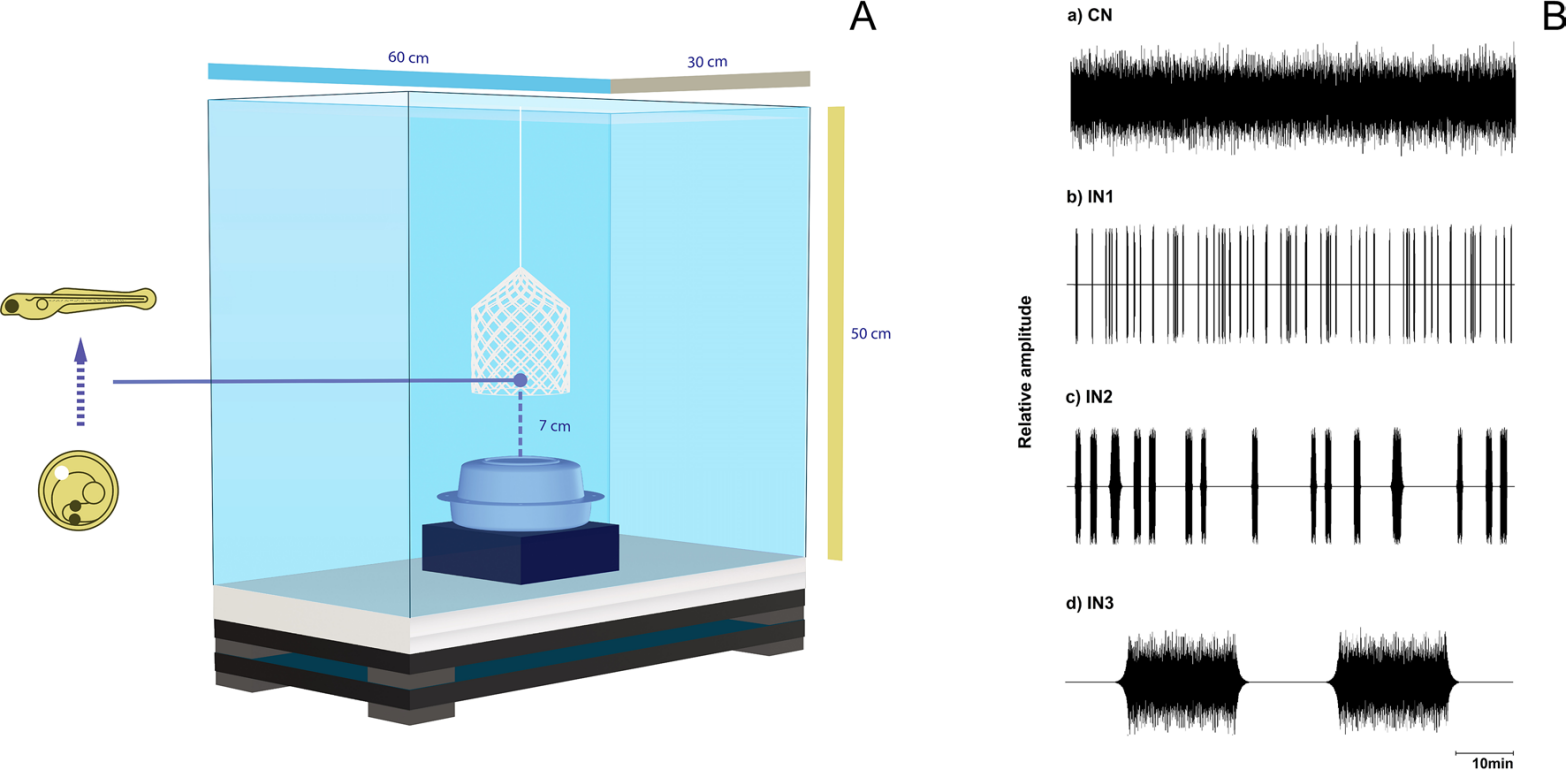
(**A**) Diagram of the acoustic treatment tank. The tank rested on top of two granite plaques separated by anti-vibratory rubber pads. Inside, a custom-made net cylinder containing zebrafish egg/larvae was suspended 7 cm above an underwater speaker (UW30, Lubel Labs, Ohio, USA) that rested on top of a polyurethane sponge. (**B**) Oscillogram of sound files used for playbacks. Control- silent conditions, CN- continuous noise at either 130 (CN_130_) or 150 dB re 1 μPa (CN_150_), IN- intermittent regime with random short noise segments (IN1): 5–12 s duration spaced by silent intervals of 1–120 s (total noise exposure of c. 15%); medium noise segments (IN2): 30–60 s interspaced by 1–10 min silence (similar noise exposure to IN1); and long noise segments (IN3) of 15 min separated by 15 min silent periods (about 50% overall noise). Illustration was created using Adobe Illustrator CC 2020 version 24.0.2 for Windows, Adobe Inc., CA, USA and Blender version 2.80 for Windows, Blender Foundation, AMS, EU.

A total of 15 experimental trials consisting of simultaneous treatments of either different noise amplitudes or varying temporal patterns (at the same noise level each) were conducted. The acoustic treatments were carried in glass tanks (60 cm length × 30 cm width × 50 cm height) equipped with top built-in illumination (~ 7000 Lux in a 12:12 light/dark cycle) and covered with a Styrofoam structure to control for light, temperature and noise conditions. No filtering system was used to avoid additional noise, but complete water changes were carried between trials to maintain appropriate water quality similar to stock conditions. Each treatment tank was mounted on top of Styrofoam boards placed over two granite slabs (1.5 cm thick) spaced by rubber pads to reduce non-controlled transmission of building vibrations. Eggs were placed inside a custom-made cylindrical fine-mesh net box (5 cm diameter, 6 cm high) suspended at ~ 7 cm above an underwater speaker (UW30, Lubel Labs, Ohio, USA) that rested on top of a sponge base to minimize transmission of playback vibrations into the tank bottom (Fig. 6A). Speakers were connected to audio amplifiers (ST-50, Ai Shang Ke, China) that were connected to laptops running Adobe Audition 3.0 for windows (Adobe Systems Inc., USA). A total of 5 experimental tanks were used alternately for the different treatments across the various trials. In “control” group, the amplifier was connected to the speaker and switched on, however there was no playback. The background noise level measured in such control condition varied between 103 and 108 dB re. 1 µPa (LZS, RMS sound level obtained with slow time and linear frequency weightings: 6.3 Hz–20 kHz).

Sound treatments consisted of white noise low-pass filtered at 1500 Hz and adjusted to the tank acoustic properties using Adobe Audition software tools to deliver a relative flat spectrum. To test for the effects of amplitude on larval zebrafish, two sound files of continuous white noise (CN) at different amplitudes or Sound Pressure Levels (SPLs), namely 130 dB (CN_130_) and 150 dB re. 1 µPa (CN_150_), were generated and played back in loop. These noise levels were similar to those found in freshwater aquatic habitats characterized by anthropogenic noise activity such as shipping^103,104^ and noise conditions in certain zebrafish housing systems^55^. To evaluate the impact of different noise temporal regimes, three additional sound files of intermittent noise and 60 min duration were generated and played back in loop at 150 dB re 1 µPa (Fig. 6B): IN1- short noise segments of 5–12 s duration spaced by silent intervals of 1–120 s (60 noise events per hour) reaching an overall noise exposure of about 15%, designed to mimic intense boat traffic noise as described in Nichols et al. (2015)^95^; IN2- medium noise segments of 30–60 s interspaced by 1–10 min silence, with 15 noise segments per hour and similar overall noise exposure to IN1; and IN3- long noise segments of 15 min separated by 15 min silent periods (about 50% overall noise), which followed a prolonged shipping activity as described by Nedelec et al. (2015)^13^ (Fig. 6B). All noise presentations contained a logarithmic fade-in and fade-out ramps of 10% of the noise presentation. We used random exposure regimes since it reflects better an acoustic environment characterized by traffic noise and it is known to cause physiological stress in fish^13,95^, and affect specifically zebrafish behaviour^105^.

Noise levels were calibrated before each experimental trial so that the intended sound level (LZS, 6.3 Hz**–**20 kHz) was reached at the bottom of the net box (~ 7 cm above the speaker) using a hydrophone (Bruel&Kjær 8104, Naerum, Denmark; frequency range: 0.1 Hz to 120 kHz, sensitivity of − 205 dB re 1 V/µPa) connected to a hand-held sound level meter (Bruel&Kjær model 2270). Additionally, the acoustic treatments were calibrated with a tri-axial accelerometer (M20-040, frequency range: 1–3 kHz, GeoSpectrum Technologies, Dartmouth, Canada). The particle acceleration sensor was placed horizontally with the acoustic center also positioned at about 7 cm from the speaker in the position occupied by the net box containing the specimens. Particle acceleration level determined for the highest sound level (150 dB re 1 µPa) in the vertical axis consisted of 120 dB/s^2^. A decrease of 20 dB in sound pressure resulted in similar variation in acceleration. The sound playbacks generated most energy in the vertical axis compared to the other orthogonal directions. The calibration was conducted using a Matlab script (paPAM) based on Nedelec et al. (2016)^106^.

### Morphological and cardiac rate measurements

The impact of the acoustic treatments on morphological development and cardiac rate of larval zebrafish was assessed based on 5 specimens per treatment in each experimental trial. The specimens were lightly sedated using low concentration (0.004%) of MS-222 buffered with sodium bicarbonate to quantify cardiac activity following previously described procedures^107^, and then observed under a stereomicroscope (Stemi 2000CS, Carl Zeiss, Jena, Germany) connected to a digital camera (Axiocam, ICc3, Carl Zeiss) and a desktop running Zen 2.3 Lite (Carl Zeiss). Photographs were taken from each specimen in lateral position for determining total body length and yolk sac size based on Teixidó et al.^108^, followed by a video of 60 s focusing on the cardiac area.

Both photographs and videos were analysed using specific tools for morphological analysis and automatic detection of cardiac activity of DanioScope software (Noldus Information Technology, Wageningen, Netherlands). The yolk sac size was defined as the lateral area (xy coordinates) delineated manually. The cardiac rate measurement was also done manually to validate the software automatic measurements and whenever the video quality was not appropriate.

The experimenter remained unaware of the treatment of the specimens under analysis (blinded experiment), since files were automatically labelled by the software with an alphanumeric code following the sequence of data acquisition. The information regarding the treatment of the specific file number was registered in a document that was subsequently used to organize information into a data sheet.

### Cortisol quantification

The analyses were conducted based on 10 larvae collected as one sample from the different treatments and for the two developmental stages analyzed. A total of eight different trials were carried to test the effect of increasing noise level, and six trials to assess the impact of different noise temporal patterns. We followed previously described procedures by Bai et al.^78^ adapted to improve extraction process. Specimens were euthanized in MS-222 300 mg/L, excess water was removed, and individuals frozen at − 80 °C in collection tubes. Prior to analysis, samples were thawed by adding 150 μl of ice-cold Millipore Ultrapure water to each tube and homogenized with a hand-held pellet mixer for 20 s. Immediately, 450 μl of pure methanol were added to each sample and vortexed at max speed for 30 s. Samples were placed at 4 °C for 1 h, vortexed again, and centrifuged at 3000 g for 10 min at 4 °C. The liquid phase was carefully collected and transferred to a new vial. The methanol extraction was again repeated following same procedure and the liquid phase collected into the same vial. Liquid nitrogen-evaporated samples were reconstituted in 500 μl of enzyme immunoassay buffer and kept at − 80 °C for less than 24 h, and vortexed prior to assay. Samples were tested in duplicate using a colorimetric cortisol enzyme immunoassay kit (Cayman Chemical, Ann Arbor, MI, USA).

### Light/dark choice assay

Larval zebrafish (5 dpf) exposed to either continuous noise (CN_150_) or control conditions were tested with the anxiety-related light/dark preference assay, which is a known behavioural paradigm to assess environmental stress in larval zebrafish^78^. Increased percentage of time spent in the light side is assumed to reflect increased anxiety in this model organism, which was previously found to prefer light or show dark avoidance (scotophobia)^109^. The light/dark preference apparatus was designed based of Bai et al.^78^ and consisted on eight individual squared transparent plastic compartments (4.0 cm length × 4.0 cm width × 3.0 cm height), each divided into two equal sized areas with distinct bottom illumination conditions—transparent/bright and opaque/dark (Fig. 4A). The apparatus was placed on top of a LED panel covered with a white translucent glass to provide uniform illumination (~ 7000 lx). The sides of the compartments were covered with black vinyl tape to control for external visual interferences. Each compartment was filled with 10 ml of system water at about 28 °C (water depth: ~ 5 mm). A single larva was carefully placed with a plastic pipette in the middle region between the two areas in each compartment and a 5 min video covering the multiple testing compartments was recorded. A total of 11 trials with simultaneous test compartments were conducted. The videos were analysed regarding the time spent in each area and a Choice Index calculated: time spent in darkness—time spent in light)/(time spent in darkness + time spent in light). Meaning that (0) reflects no preference, (− 1) larva spent all the time swimming in the light zone, and (1) all the time in the dark zone (Fig. 4B).

### Spontaneous alternation behaviour assay

Larval zebrafish were further tested regarding the Spontaneous Alternation Behaviour (SAB), which describes the tendency of animals to alternate their turn direction in consecutive turns while navigating through the environment^56,110^. Each choice statistically depends on the previous one but, unlike other memory assessment paradigms, SAB does not require prior training or reinforcement. Moreover, the first occurrence of SAB is tightly correlated with the development of the hippocampus in several vertebrates^56,111,112^, which further evidences its mnestic origin.

The SAB was assessed in a 3D printed T-maze designed based on Bögli and Huang^113^ and consisted of two starting arms converging into a main arm that bifurcated in the end into two goal arms, each leading to a goal pool (Fig. 5A). Starting and main arms had a length of 5.0 cm, while goal arms measured 2.5 cm. All arms had a width of 0.5 cm and depth of 1.0 cm, and the goal arenas were 19.50 cm^2^. The maze was illuminated from below by placing it on top of a LED panel**.** For each of the 9 trials, 10 larvae were tested simultaneously. The maze was prefilled with fresh system water at 28 °C (to a depth of about 0.7–0.75 cm) and the intersections between the two starting arms and the main arm were both initially closed with plastic tubes. Testing larvae were carefully placed simultaneously using a plastic pipette in one of the starting arms and, after 5 min of acclimation, the respective tube was removed to start the trial. Successful entry was counted when a larva fully entered one of the goal pools within the 10 min of the recording. In the case of returning to the main arm and/or entering the second goal pool, only the first entry was counted. In order to evaluate potential changes in motor behaviour due to acoustic stress, another set of specimens subject to the same treatments/control (10–15 per trial) were analysed regarding the total swimming area in a petri dish (8 cm diameter) based on video analysis (5 min). The swimming area of each individual was determined using a grid (5x5 mm) and based on the total number of squares covered.

### Data analysis

Comparisons of hatching rate, total length, mortality, cardiac rate, yolk sac size and cortisol levels between groups exposed to different noise levels and temporal patterns variations were based on One-way ANOVA tests. These were followed by LSD multiple comparison post hoc tests to verify pairwise differences. The relationship between cardiac rate and yolk sac size was further assessed with a Pearson’s correlation coefficient. Additionally, given that control and continuous noise (CN or CN_150_) conditions were the same in both experiments (testing the effects of different noise levels and varying temporal patterns) and results were not statistically different, the data collected in both experiments were merged and used for testing both effects.

The potential effects of the batch quality/trial were assessed through mixed models containing “batch” as a random factor, and no interaction between these variables was detected.

Differences in light/dark preference were quantified based on a Choice Index and compared between treatment groups based on a two-tailed Man-Whitney U test. Finally, the presence of spontaneous alternation behaviour and the total swimming area were compared between noise-exposed and control groups using 2-tailed t-tests.

The assumptions for parametric analyses were confirmed through the inspection of normal probability plots and by performing the Levene’s test for homogeneity of variances. All statistical tests were performed using SPSS v26 (IBM Corp. Armonk, NY, US) and Statistica 10 for Windows (Dell Software, Inc., Round Rock, T, USA).

Illustrations were created using Adobe Illustrator CC 2020 version 24.0.2 for Windows, Adobe Inc., CA, USA and Blender version 2.80 for Windows, Blender Foundation, AMS, EU. Graphs were created using GraphPad Prism version 8.0.1 for Windows, GraphPad Software, CA, USA.
